# Genetic variation and dopamine D2 receptor availability: a systematic review and meta-analysis of human *in vivo* molecular imaging studies

**DOI:** 10.1038/tp.2016.22

**Published:** 2016-03-01

**Authors:** B S Gluskin, B J Mickey

**Affiliations:** 1 Department of Psychiatry, University of Michigan Medical School, Ann Arbor, MI, USA; 2 School of Kinesiology, University of Michigan, Ann Arbor, MI, USA; 3 Department of Psychiatry, University of Utah School of Medicine, Salt Lake City, UT, USA

## Abstract

The D2 dopamine receptor mediates neuropsychiatric symptoms and is a target of pharmacotherapy. Inter-individual variation of D2 receptor density is thought to influence disease risk and pharmacological response. Numerous molecular imaging studies have tested whether common genetic variants influence D2 receptor binding potential (BP) in humans, but demonstration of robust effects has been limited by small sample sizes. We performed a systematic search of published human *in vivo* molecular imaging studies to estimate effect sizes of common genetic variants on striatal D2 receptor BP. We identified 21 studies examining 19 variants in 11 genes. The most commonly studied variant was a single-nucleotide polymorphism in *ANKK1* (rs1800497, Glu713Lys, also called ‘Taq1A'). Fixed- and random-effects meta-analyses of this variant (5 studies, 194 subjects total) revealed that striatal BP was significantly and robustly lower among carriers of the minor allele (Lys713) relative to major allele homozygotes. The weighted standardized mean difference was −0.57 under the fixed-effect model (95% confidence interval=(−0.87, −0.27), *P*=0.0002). The normal relationship between rs1800497 and BP was not apparent among subjects with neuropsychiatric diseases. Significant associations with baseline striatal D2 receptor BP have been reported for four *DRD2* variants (rs1079597, rs1076560, rs6277 and rs1799732) and a *PER2* repeat polymorphism, but none have yet been tested in more than two independent samples. Our findings resolve apparent discrepancies in the literature and establish that rs1800497 robustly influences striatal D2 receptor availability. This genetic variant is likely to contribute to important individual differences in human striatal function, neuropsychiatric disease risk and pharmacological response.

## Introduction

The dopamine D2 receptor is a G-protein-coupled receptor that is highly expressed in the striatum. D2 receptors mediate an array of fundamental brain functions, including reward behavior, regulation of movement, learning and memory, and attention. The D2 receptor is also important clinically as a target of pharmacotherapy for psychosis, parkinsonism, brain injuries and restless legs syndrome.^1^ These diverse roles attest to the clinical and neurobiological importance of the D2 receptor.

Substantial inter-individual variation in D2 receptor expression has been observed in human postmortem and *in vivo* imaging studies, with typical coefficients of variation in the range of 20–60%.^2, 3, 4, 5, 6, 7^ Individual differences in D2 receptor expression are hypothesized to contribute to differences in motivated behaviors and risk of related neuropsychiatric disorders. For example, striatal D2 receptor manipulation in rodents alters motivated behaviors,^8^ and human studies have consistently demonstrated abnormally low striatal D2 receptor levels among individuals with addictions.^9^ The factors that underlie individual differences in D2 receptor expression in humans are not yet defined, but presumably both environmental and genetic factors are at work.

To address the role of genetic factors, numerous studies have used *in vivo* molecular imaging (positron emission tomography (PET) or single-photon emission computed tomography (SPECT)) to test whether common genetic variants influence D2 receptor binding potential (BP) in humans. However, consistent and robust genetic effects have not yet been demonstrated, and the magnitude of genetic effects is unknown. For example, among seven published studies of the single-nucleotide polymorphism (SNP) rs1800497, four^4, 6, 10, 11^ reported a significant association and three^5, 12, 13^ found no significant effect on striatal BP. There are several potential causes of such discrepancies. A genetic variant may have no true underlying effect (type I error), the true effects may be different in different studies (heterogeneity), or a true effect may be present but not consistently detected due to the low power of individual studies (type II error). This latter possibility may be particularly relevant to molecular imaging studies as their cost and complexity often necessitate small sample sizes.

Recent work has highlighted the importance of resolving such apparent discrepancies in the genetics-imaging literature. For example, despite compelling preclinical evidence and numerous human imaging studies, a recent meta-analysis of the brain-derived neurotrophic factor Val66Met polymorphism found no significant effect on hippocampal volume.^14^ Similarly, meta-analyses of the variable repeat polymorphism in the promoter of the serotonin transporter and amygdala activation have indicated a much smaller effect size than initially reported.^15, 16^ Our objective here was to perform a review and quantitative analysis of all published studies that tested genetic polymorphisms and measured human *in vivo* D2 receptor BP, thereby establishing associations that are known with confidence.

## Materials and methods

We performed a systematic search of PubMed in May 2015 using the following search string: ‘(variant* OR polymorphism* OR genetic OR gene) AND (pet OR positron OR emission OR spect OR photon OR tomography) AND (dopamine OR d2 OR d3) AND receptor.' The titles and abstracts of all search results were reviewed to identify studies that examined genetic polymorphisms and used human D2 molecular imaging (PET or SPECT). Full text was retrieved for all potentially relevant articles. The references of full text articles were scanned to identify additional sources missed by the original search.

Meta-analysis was performed when possible for genetic variants tested in multiple independent samples. We used the ‘metafor' package (ver. 1.9–3, W. Viechtbauer, http://cran.r-project.org/web/packages/metafor) and ‘meta' package (ver. 3.7-0, G. Schwarzer, http://cran.r-project.org/web/packages/meta) with RStudio (ver. 0.97.551) within the R statistical computing environment (ver. 3.0.2, http://www.R-project.org/). The effect size was represented by the standardized mean difference (SMD), that is, the mean BP for one genotype group minus mean BP for the second genotype group, divided by Hedges' pooled s.d. Inverse-variance weighting was used to compute the pooled SMD. Fixed-effect and random-effects models were estimated. Forest plots and funnel plots were inspected for outliers and bias. Sensitivity analyses used the leave-one-out method to test for undue influence of single studies. When Cochran's *Q*-test for heterogeneity indicated nonsignificant heterogeneity across studies (*I*^2^<0.5 and *P*>0.10 as suggested^17^), we adopted the effect size from the fixed-effect model as our final estimate. Only BP values from anatomically defined regions of interest were used in meta-analyses. Because BP estimates from different brain regions within the same sample are highly correlated, and valid meta-analysis requires independent samples, a single estimate from each genotype group was derived (pooled mean and s.d. with inverse-variance weighting) in cases where BP estimates from multiple anatomical regions were reported. Bilateral striatum was preferred and was used for all studies except Savitz *et al.*^11^ who reported a middle caudate region of interest. To avoid the confounding effects of disease and treatment, medication-free healthy control subjects were analyzed separately from subjects with neuropsychiatric disorders. Studies with different demographic characteristics (sex and age distribution) and imaging methods (SPECT versus PET, region of interest imaged) were first analyzed together based on the assumption that these study-level variables have effects on binding measures that are similar in the two genotype groups. Although the small number of studies severely limited our power to detect statistical effects of these study-level variables on the estimated genetic effect, we did explore these potential moderators with fixed-effect meta-regression and leave-one-out sensitivity analysis.

## Results

**Figure 1:**
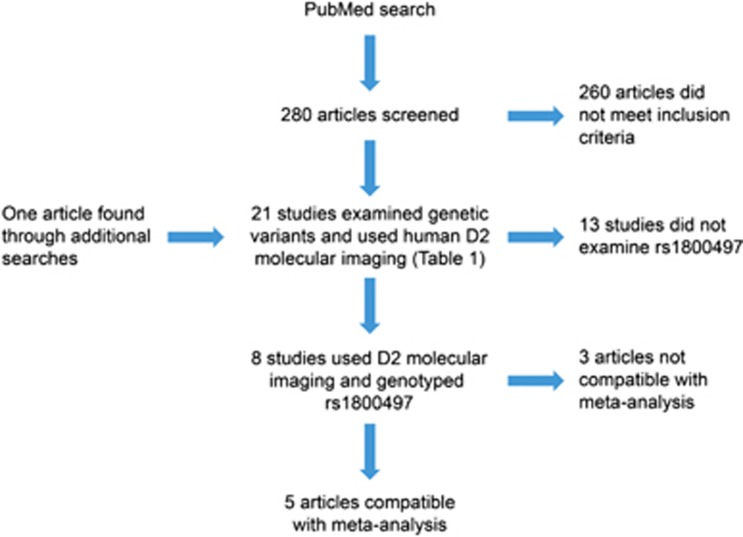
Flow diagram of systematic literature search.

We identified 21 studies that examined genetic polymorphisms and used human D2 molecular imaging (Figure 1). The 21 studies examined 19 variants in 11 genes (Table 1). None of the investigated variants were in strong linkage disequilibrium (LD; all *r*^2^<0.8) except rs1079597 and rs1076560 (*r*^2^=0.87).

The most commonly studied variant was rs1800497, a SNP within the ankyrin repeat and kinase domain containing 1 (*ANKK1*) gene and 10 kb downstream from the dopamine D2 receptor (*DRD2*) gene. This missense mutation (C>T) is predicted to change a glutamate residue to a lysine residue at position 713 (Glu713Lys) within the 11th ankyrin repeat of the ANKK1 protein.^33^ This variant is often referred to as the ‘Taq1A' polymorphism (after a restriction enzyme initially used to detect it) with the minor Lys713 allele denoted ‘A1' and the major Glu713 allele denoted ‘A2'.^34^

**Figure 2:**
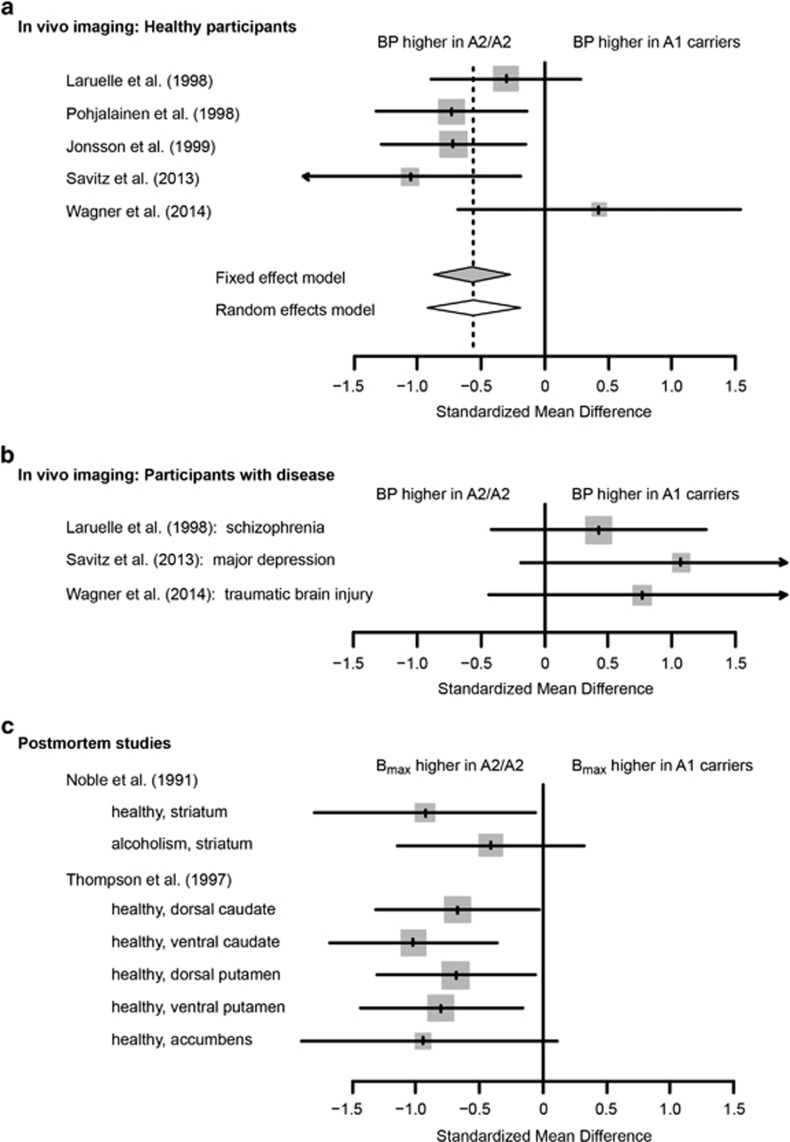
Forest plots of effect size for rs1800497 (Taq1A) and D2 receptor binding. Each genotype group comparison is represented by a gray square and horizontal error bars (mean and 95% confidence intervals). Square size is proportional to study weight. The solid vertical line represents the null hypothesis (no effect of the genetic variant). (**a**) *In vivo* imaging studies of healthy participants. Diamonds represent means and 95% confidence intervals for the fixed-effect model (filled diamond) and random-effects model (open diamond). The dashed vertical line is the fixed-effect weighted mean. (**b**) *In vivo* imaging studies of participants with disease. (**c**) Postmortem studies. BP, binding potential; *B*_max_, total number of receptors.

Five of the eight Taq1A studies were compatible with meta-analysis. One article was not compatible because baseline BP values were not reported.^12^ A second article was incompatible because only extrastriatal BP was quantified.^18^ A third study^10^ was excluded because it included subjects from a previously reported sample.^6^ The remaining five studies included 194 healthy participants. Striatal BP was significantly and robustly lower among healthy A1-allele carriers relative to healthy A2 homozygotes (Figure 2a). The weighted SMD was −0.57 (95% confidence interval (95% CI)=(−0.87, −0.27), *P*=0.0002) under the fixed-effect model and −0.56 (95% CI=(−0.93, −0.19), *P*=0.003) under the random-effects model. Sensitivity analysis showed that the results were robust when individual studies were omitted (all *P*<0.005) indicating that no single study unduly influenced the results. We found no evidence of heterogeneity across studies (*Q*=5.7, degrees of freedom (df)=4, *I*^2^=0.29, *P*=0.22). Likewise, a funnel plot did not suggest heterogeneity or bias (data not shown). Thus, our results are consistent with a single underlying effect of the Taq1A polymorphism across studies.

Meta-regression and sensitivity analyses were used to explore the potential influence of study imaging modality (PET versus SPECT), brain region of interest, sex distribution and mean age on the Taq1A findings. We considered these exploratory analyses, as our power to detect effects of study-level variables across five studies was very limited. Meta-regression suggested that sex distribution may be a moderator of the Taq1A effect (*β*=0.024, s.e.m.=0.011, *Q*=4.4, df=1, *P*=0.035) with stronger genetic effects evident in studies with a greater proportion of females. Meta-regression revealed no significant effects of imaging modality (*P*=0.48), brain region (*P*=0.27) or mean subject age (*P*=0.38). Finally, fixed-effect analyses that excluded the SPECT study^5^ or the study that quantified a middle caudate region^11^ produced similar estimates of effect size (SMD=−0.66 and −0.50, *P*=0.0002 and 0.002, respectively).

Three of the Taq1A *in vivo* imaging studies^5, 11, 13^ quantified striatal BP in participants with disease. Laruelle *et al.*^5^ reported data from 23 subjects with schizophrenia, Savitz *et al.*^11^ reported data from 12 subjects with major depressive disorder and Wagner *et al.*^13^ reported data from 12 subjects with traumatic brain injury. In all cases, participants were free of dopaminergic medications for at least 3 weeks, and individuals with recent alcohol or drug dependence were excluded. In contrast to healthy participants, striatal BP in these subjects tended to be higher (rather than lower) among A1-allele carriers relative to A2 homozygotes (Figure 2b). The effect sizes for each of these three patient groups were not significantly different from 0, but they did differ significantly from the above-calculated weighted mean effect size for healthy controls (95% CI for between-group differences: (0.1, 1.9), (0.4, 2.9) and (0.1, 2.6), respectively). This finding suggested that for each of these diagnostic groups, the disease moderated the effect of the Taq1A polymorphism on D2 receptor BP. Meta-regression including healthy and disease samples confirmed that disease status was a significant moderator (*Q*=12.0, df=1, *P*=0.0005).

In addition to the eight *in vivo* imaging studies, two studies of postmortem brain have reported binding density (*B*_max_) versus Taq1A status,^2, 3^ so we extracted effect sizes from these studies as well. Striatal binding was generally lower among healthy A1-allele carriers relative to A2 homozygotes (Figure 2c) and the magnitude of this difference was consistent with *in vivo* measurements from healthy participants (Figure 2a).

The intronic *DRD2* SNP rs1079597 was examined in two comparable, independent, molecular imaging studies.^4, 5^ This variant, which is in moderate LD with the Taq1A variant (*r*^2^=0.70), is sometimes called the ‘Taq1B' polymorphism, with the minor allele denoted ‘B1' and the major allele denoted ‘B2'.^35, 36^ The first study^5^ reported no significant effect of this polymorphism on D2 binding but the second study^4^ reported significantly lower striatal BP among healthy B1-allele carriers relative to healthy B2 homozygotes. Although this apparent discrepancy has been interpreted as a replication failure,^4^ the effect sizes are actually consistent with each other (mean=−0.40, 95% CI=(−1.02, 0.21); and mean=−0.72, 95% CI=(−1.30, 0.15), respectively) and the weighted mean effect size from the two samples is −0.58 (95% CI=(−1.00, −0.15), *P*=0.008). Thus, the Taq1A and Taq1B polymorphisms appear to have a similar magnitude of association with D2 receptor BP in healthy participants.

Catechol-*O*-methyltransferase (COMT) is an enzyme that degrades dopamine, so genetic variation in the *COMT* gene has been hypothesized to affect dopamine receptor availability. The effect of the *COMT* missense variant rs4680 (Val158Met) on striatal D2 receptor BP was examined in three independent molecular imaging studies. One study of 45 cigarette smokers found no significant effect, but reported only a lower bound on significance (*P*>0.25).^12^ A second study of 45 healthy participants also reported no significant effect (SMD=0.02, 95% CI=(−0.59, 0.63)).^19^ A third study of 15 individuals with 22q11 deletion syndrome reported lower binding associated with the Met158 allele (SMD=1.38, 95% CI=(0.17, 2.60)).^20^ Thus, the available evidence does not support an effect of this polymorphism on D2 receptor BP in diploid individuals, but the genetic effects may be stronger among individuals with 22q11 deletion syndrome, who have only one copy of *COMT*.

Two independent molecular imaging studies examined a 3′ variable-number tandem repeat polymorphism in the dopamine transporter gene (*SLC6A3*). No significant differences between genotype groups were found among healthy controls^13^ or cigarette smokers.^12^ Among patients with traumatic brain injury, 10-repeat homozygotes showed marginally higher D2 receptor binding than 9-repeat carriers (*P*=0.052).^13^

For the remaining 15 genetic variants identified in the literature search, we found no other replications in which two or more comparable, independent, studies of striatal D2 receptors tested the same variant. Significant associations with baseline (resting) striatal D2 receptor BP were reported for 4 of the 15 variants. Three of the 4 variants reported were in the *DRD2* gene: the intronic SNP rs1076560 (ref. 21), the synonymous SNP rs6277 (ref. 23) and the upstream single-nucleotide insertion rs1799732 (ref. 4). Finally, Shumay *et al.* studied an intronic variable-number tandem repeat polymorphism in the circadian gene *PER2* based on preclinical evidence linking striatal dopamine release with circadian rhythms and *PER2*. They reported that fewer repeats were associated with lower baseline D2 receptor BP.^32^

Several genetic association studies used the radiotracer [^11^C] raclopride, which is displaceable by endogenous dopamine, in combination with a behavioral challenge intended to induce striatal dopamine release (cigarettes, pain and reward task). Significant associations were reported for polymorphisms in genes directly involved in dopamine signaling, viz., the dopamine transporter,^12^ D2 receptor,^26^ D3 receptor^27^ and D4 receptor.^12^ In addition, significant associations were found for variants in genes for the serotonin 2C receptor^28^ and mu-opioid receptor^30, 37^—both known to regulate dopamine release via their expression in ventral tegmental interneurons—and for variants in the genes for leptin^29^ and oxytocin,^31^ which act at their respective receptors within the mesoaccumbal and nigrostriatal pathways.

## Discussion

This systematic review and meta-analysis has produced several principal findings. First, variants in *DRD2* and 10 other genes have been tested for effects on *in vivo* striatal D2 receptor BP, but only 4 variants (rs1800497, rs1079597, rs4680 and *SLC6A3* variable-number tandem repeat) have been examined in two or more comparable, independent samples. Second, despite apparent discrepancies in the literature, the *ANKK1* Taq1A polymorphism (rs1800497) is robustly associated with D2 receptor BP in healthy humans. Third, the *DRD2* variant rs1079597 has effects on D2 receptor BP that are similar to rs1800497. Fourth, several examples were identified in which the presence of a neuropsychiatric syndrome modified the effects of genetic variants on D2 receptor BP.

Our study has notable limitations. The first type of limitation is related to interpretation of *in vivo* receptor binding measures. The PET and SPECT studies examined here estimated a quantity proportional to *B*_max_/*K*_d_, where *B*_max_ is the total number of available receptor sites and 1/*K*_d_ is the affinity of the radiotracer for the receptor. Consequently, group differences may arise from a difference in *B*_max_, a difference in *K*_d_ or both. In addition, because raclopride is displaceable by endogenous dopamine *in vivo*, *B*_max_ reflects the number of receptor sites not occupied by endogenous ligand rather than the total number of receptor sites.^38^ In other words, BP is sensitive to endogenous dopaminergic tone, which may be an additional source of variance. Furthermore, raclopride is known to bind D2 receptors in both the G-protein-coupled (‘high' or active) state and the non-coupled (‘low' or inactive) state, so genetic influences on the proportion of receptors in the high versus low state cannot be distinguished.^39^ It is notable that different tracers or modeling methods used across different laboratories will yield different absolute BP estimates (for example, SPECT versus PET, or reference region versus arterial input function). BP estimates may also be altered by between-study differences in how the striatum was defined anatomically, and by striatal atrophy in disease groups. We believe that such specific methodological factors are unlikely to substantially influence our analyses of genetic effect sizes because such factors are likely to influence genotype groups equally.

The second type of limitation is related to systematic review and meta-analysis methodology. With the exception of rs1800497, the paucity of replications prevented reliable quantification of effect size for most genetic variants. In several cases, studies could not be included in meta-analyses because effect sizes (or data sufficient to compute effect sizes) were not reported. We used heterogeneity tests, funnel plots and sensitivity analyses to evaluate for bias and inconsistency across studies, but the utility of these approaches is limited when examining only five studies. Meta-analysis methodology is incapable of answering questions about (or controlling for) potentially interesting individual-level variables such as sex, age, race/ancestry, body mass index or striatal anatomy. Therefore, we were unable to determine with confidence whether these variables moderated the effect of rs1800497 on *in vivo* D2 receptor binding. If protocols for sharing of molecular imaging data are developed, future mega-analysis^40^ of individual participant data could address these and other interesting questions. Finally, relevant data may have been unreported due to negative-results reporting bias (that is, the ‘file drawer problem') and our meta-analysis may have overlooked relevant articles.

Our most robust finding is the effect of the *ANKK1* Taq1A variant (rs1800497) on striatal BP in healthy participants. BP was lower among carriers of the minor allele (‘A1' or Lys713) relative to A2 homozygotes, with a SMD of −0.57 and 95% CI of (−0.87, −0.27). Furthermore, the findings from *in vivo* molecular imaging studies were consistent with effect sizes for *B*_max_ values measured in two postmortem studies. It is instructive to compare this effect size of 0.57 with other, commonly encountered, individual differences. Human males are typically taller and heavier than females, but the sexes overlap substantially. Among US adults, the SMD in height between men and women is ~1.2 and that for weight is ~0.4,^41^ so we can conclude that the Taq1A polymorphism is more strongly associated with D2 receptor BP than sex is with weight, but less so than sex is with height. It is also instructive to consider the proportion of the observed variance in BP that is explained by the Taq1A variant, as there are presumably numerous genetic and environmental factors that influence D2 receptor BP. For a SMD *d* with group sizes *n*_1_ and *n*_2_, the equivalent Pearson correlation is *r*=*d*/(*d*^2^+*c*)^1/2^, where *c*=(*n*_1_+*n*_2_)^2^/*n*_1_*n*_2_ (ref. 17). In the case of the Taq1A variant, *d*=0.57, *n*_1_=73 and *n*_2_=121, so the equivalent *r*=0.266 and *r*^2^=0.071. This indicates that the proportion of variance in striatal BP that is explained by rs1800497 is ~7%, with a 95% CI of (1.7%, 15%). With an effect size of this magnitude, it is not surprising that some molecular imaging studies did not detect an effect of the Taq1A variant on D2 receptor BP. The largest study reviewed here included just 56 healthy participants. This sample size has only 50–60% power to detect a true effect size of 0.57 (two-tailed test, *α*=0.05), whereas a sample of ~200 subjects provides >95% power.^42^

Several large studies have reported associations of the A1 allele of rs1800497 with poorer cognitive function. A study of ~2000 older adults linked the A1 allele with lower general cognitive ability^43^ and a study of ~500 patients linked the A1 allele with poorer cognitive outcomes after traumatic brain injury.^44^ A recent meta-analysis suggested an association of the A1 allele with attention deficit hyperactivity disorder, although the authors observed significant unexplained heterogeneity across studies,^45^ possibly related to differences in diagnostic procedures or clinical subtypes. A recent study of ~1300 adolescents showed that A1-allele carriers had poorer performance on visuospatial working memory tasks.^46^ That study also measured striatal activation using functional magnetic resonance imaging during a reward task and found no significant main effects of the Taq1A polymorphism, but the authors did detect an interaction whereby working memory performance was associated with striatal activation only among A1 carriers.^46^ This finding is consistent with the idea that lower D2 receptor expression among A1 carriers increases the dependence of working memory performance on striatum-based motivational circuitry.^47^ Taken together, these studies suggest that lower expression of D2 receptors among A1 carriers confers poorer cognitive function, although this model is yet to be directly tested.

Although the association of rs1800497 with neurocognitive function has been well studied, remarkably little is known about the impact of this variant at the molecular, cellular or circuit level. This SNP is predicted to cause a missense mutation in *ANKK1* (Glu713Lys) and lies within 10 kb of *DRD2*.^33^ To our knowledge, no *in vitro* mechanistic studies have yet examined the effects of rs1800497 on the expression or molecular function of DRD2 or ANKK1 proteins. ANKK1 and DRD2 mRNA are both expressed in human striatum (Allen Human Brain Atlas, http://human.brain-map.org),^48^ and expression of ANKK1 in rodents appears to be regulated by dopamine.^49, 50^ To our knowledge, direct regulation of D2 receptors by ANKK1 has not been shown. Although a causal link between rs1800497 and D2 receptor density or affinity appears plausible, it is equally plausible that another polymorphism in LD with rs1800497 is the causal variant. For example, rs1800497 is in LD with the *ANKK1* missense SNP rs7118900, which is predicted to create a new ANKK1 phosphorylation site and which has been associated with differential subcellular ANKK1 expression patterns *in vitro*.^49, 51^ It is also in LD with the intronic *DRD2* SNP rs1079597 (described above) and with the intronic *DRD2* SNPs rs2283265 and rs1076560, which were reported to alter DRD2 RNA splicing.^52^ Given the clear association of rs1800497 with D2 receptor BP in humans that we have demonstrated here, and the multiple potentially causal SNPs in LD with rs1800497, further *in vitro* and *in vivo* mechanistic studies are warranted.

Our analyses suggest that disease status and sex may moderate the genetic effect of rs1800497 on D2 receptor BP. The robust association of the minor allele with lower D2 receptor BP that we observed among healthy subjects was not present in samples with schizophrenia, depression or traumatic brain injury (TBI) (Figure 2b). Similarly, exploratory analysis suggested that the genetic effect of rs1800497 was weaker in studies that included a greater proportion of males. Similar disease-by-genotype and sex-by-genotype interactions have been reported in cognitive outcomes after TBI. The rs1800497 minor allele was associated with poorer cognition among predominantly mild TBI^44^ but with better cognition among a more severe TBI sample.^53^ Along the same lines, rs1800497 was a significant predictor of cognitive performance among females with TBI, but not among males with TBI.^54^ Taken together with our finding that the effect of rs1800497 on D2 receptor BP is stronger in studies with a greater proportion of females, these findings suggest that this polymorphism may be more penetrant in females. Although the neurobiological basis of this sex difference is unclear, sex steroids may have a role, given evidence in rodents that estrogens and testosterone influence D2 receptor expression.^55, 56, 57^

Our findings carry implications for future studies of the D2 receptor. First, based on the robust evidence that the ANKK1 polymorphism rs1800497 is associated with D2 receptor binding in humans, future studies are warranted to determine whether rs1800497 is the causal variant (versus another polymorphism in LD), and to elucidate the molecular and cellular basis of this association. Second, our findings highlight the possibility that other variants of similar effect size exist. Adequately powered human *in vivo* molecular imaging studies are warranted to identify the most influential variants. Third, genetic variation, sex and disease may interact to influence D2 receptor BP in complex ways, so the design and analysis of future studies that examine any one of these variables should consider effects of the other two as well. Such effects could be especially important for sexually dimorphic diseases (for example, depression and attention deficit hyperactivity disorder). Our findings also raise the possibility of interactions with other variables known to influence D2 receptor BP (for example, age and body mass index). Ultimately, we expect that a fuller description of genetic and environmental factors that influence D2 receptor levels and related neurocognitive functions will improve our understanding of neuropsychiatric disease risk, pharmacological response and clinically relevant outcomes.

## Figures and Tables

**Table 1:** Published studies of genetic polymorphisms and *in vivo* D2 receptor BP

*Study*	*No. of subjects*	*Age, years, mean (s.d.)*	*Sex (% female)*	*Imaging technique*	*Reported effects on striatal D2 receptor BP in vivo*
ANKK1 *rs1800497 Glu713Lys ‘Taq1A' (missense)*					
Laruelle *et al.*^5^	47 23^a^	NA	14	[^123^I] IBZM SPECT	No significant difference between genotype groups; no significant difference between healthy and schizophrenic participants
Pohjalainen *et al.*^6^	54	40 (17)	39	[^11^C] RAC PET	A1 carriers significantly lower than A2/A2
Jönsson *et al.*^4^	56	32 (12)	23	[^11^C] RAC PET	A1 carriers significantly lower than A2/A2
Brody *et al.*^12^	45	37 (11)	29	[^11^C] RAC PET	No significant difference between genotype groups; no significant association with cigarette-induced decrease in binding
Hirvonen *et al.*^10^	45	36 (14)	40	[^11^C] RAC PET	A1 carriers significantly lower than A2/A2; subsample of Pohjalainen *et al.*^6^
Hirvonen *et al.*^18^	38	29 (7)	0	[^11^C] FLB 457 PET	A1 carriers significantly higher than A2/A2 in cerebral cortex and thalamus (extrastriatal)
Savitz *et al.*^11^	24 12^b^	35 (8) 38 (11)^b^	54 67^b^	[^11^C] RAC PET	A1 carriers significantly lower than A2/A2 among healthy controls; A1 carriers significantly higher than A2/A2 among depressed patients; no significant association with reward-related decrease in binding
Wagner *et al.*^13^	13 12^c^	~30 ~33	0	[^11^C] RAC PET	No significant difference between genotype groups for either healthy controls or patients with traumatic brain injury
					
COMT *rs4680 Val158Met (missense)*					
Brody *et al.*^12^	45	37 (11)	29	[^11^C] RAC PET	No significant difference between genotype groups for baseline binding; significantly greater cigarette-induced decrease in binding among Val/Val subjects relative to Met carriers
Hirvonen *et al.*^19^	45	36 (14)	40	[^11^C] RAC PET	No significant difference between genotype groups
Hirvonen *et al.*^19^	38	29 (7)	0	[^11^C] FLB 457 PET	No significant difference between genotype groups (extrastriatal)
Boot *et al.*^20^	15^d^	NA	60^d^	[^123^I] IBZM SPECT	Met hemizygotes significantly lower than Val hemizygotes (all participants with 22q11 deletion syndrome)
					
DAT *(SLC6A3) 40-bp VNTR polymorphism (3′ untranslated region)*					
Brody *et al.*^12^	45	37 (11)	29	[^11^C] RAC PET	No significant difference between genotype groups for baseline binding; significantly greater cigarette-induced decrease in binding among 9-repeat allele carriers compared with 10-repeat homozygotes
Wagner *et al.*^13^	13 12^c^	NA	0	[^11^C] RAC PET	No significant difference between genotype groups in healthy controls; 10-repeat homozygotes marginally higher than 9-repeat carriers among patients with traumatic brain injury
					
DRD2 *rs1079597*^e^ *‘Taq1B' (intronic)*					
Laruelle *et al.*^5^	47 23^a^	NA	14	[^123^I] IBZM SPECT	No significant difference between genotype groups; no significant difference between healthy and schizophrenic participants
Jönsson *et al.*^4^	56	32 (12)	23	[^11^C] RAC PET	B1 carriers significantly lower than B2/B2
					
DRD2 *rs1076560*^e^ *G>T (intronic)*					
Bertolino *et al.*^21^	32	NA	NA	[^123^I] IBZM SPECT	T-allele carriers significantly lower than G/G; binding values not reported
Taurisano *et al.*^22^	26	23 (3)	50	[^123^I] IBZM SPECT	Main effect of genotype not reported; significant interaction between genotype and schizotypy; binding values not reported
					
DRD2 *rs6277 C957T (synonymous)*					
Hirvonen *et al.*^23, 24^	45	36 (14)	40	[^11^C] RAC PET	T allele associated with significantly higher BP
Hirvonen *et al.*^10^	45	36 (14)	40	[^11^C] RAC PET	T allele associated with significantly lower affinity (*K*_d_); no significant association with receptor density (*B*_max_); subsample of Hirvonen *et al.*^23, 24^
Hirvonen *et al.*^18^	38	29 (7)	0	[^11^C] FLB 457 PET	T allele associated with significantly lower BP in cerebral cortex and thalamus (extrastriatal)
					
DRD2 *rs1801028 Ser311Cys (missense)*					
Pohjalainen *et al.*^25^	49	41 (18)	33	[^11^C] RAC PET	No significant difference between genotype groups
					
DRD2 *rs1799732 −141C ins/del (upstream)*					
Jönsson *et al.*^4^	56	32 (12)	23	[^11^C] RAC PET	Deletion associated with significantly higher binding
Hirvonen *et al.*^18^	38	29 (7)	0	[^11^C] FLB 457 PET	No significant difference between genotype groups (only extrastriatal regions measured)
					
					
DRD2 *(CA)*_*n*_ *repeat polymorphism (intron 2)*					
Jönsson *et al.*^4^	56	32 (12)	23	[^11^C] RAC PET	No significant difference between genotype groups
					
DRD2 *rs4274224 (intronic) and rs12364283 (upstream)*					
Peciña *et al.*^26^	52	27 (5)	58	[^11^C] RAC PET	No significant difference between genotype groups for baseline binding for either variant; significantly lower pain-induced decrease in binding among rs4274224 A/G heterozygotes relative to A/A or G/G homozygotes
					
DRD3 *rs6280 Ser9Gly (missense)*					
Savitz *et al.*^27^	26 10^b^	34 (8) 38 (11)^b^	58 80^b^	[^11^C] RAC PET	No significant difference between genotype groups for baseline binding; Gly allele associated with significantly greater reward-induced decrease in binding
					
DRD4 *48-bp VNTR polymorphism (exon 3)*					
Brody *et al.*^12^	45	37 (11)	29	[^11^C] RAC PET	No significant difference between genotype groups for baseline binding; significantly greater cigarette-induced decrease in binding among subjects with fewer than seven repeats
					
HTR2C *rs6318 Cys23Ser (missense)*					
Mickey *et al.*^28^	54	27 (5)	60	[^11^C] RAC PET	No significant difference between genotype groups for baseline binding; significantly greater pain-induced decrease in binding among Ser allele carriers
					
LEP *rs12706832 (intronic) and rs3828942 (intronic)*					
Burghardt *et al.*^29^	50	26 (5)	56	[^11^C] RAC PET	No significant difference between genotype groups for baseline binding for either variant; significantly greater pain-induced decrease in binding among rs12706832 G/G homozygotes
					
OPRM1 *rs1799971 A118G Asn40Asp (missense)*					
Domino *et al.*^30^	20^f^	26 (5)^f^	0^f^	[^11^C] RAC PET	Differences between genotype groups not reported for baseline binding; significantly greater cigarette-induced decrease in binding among G-allele carriers relative to A/A homozygotes
					
OXT *rs4813625 (upstream)*					
Love *et al.*^31^	55	27 (5)	58	[^11^C] RAC PET	No significant difference between genotype groups for baseline binding; among females only, significantly greater pain-induced decrease in binding among C-allele carriers compared with G/G
					
PER2 *VNTR polymorphism (intron 3)*					
Shumay *et al.*^32^	52	35 (9)	17	[^11^C] RAC PET	Carriers of 3-repeat or rare alleles significantly lower than 4-repeat homozygotes

Abbreviations: BP, binding potential; bp, base pair; FLB 457, (S)-5-bromo-N-((1-ethyl-2-pyrrolidinyl)methyl)-2,3-dimethoxybenzamide; IBZM, iodobenzamide; NA, not available; PET, positron emission tomography; RAC, raclopride; SPECT, single-photon emission computed tomography; VNTR, variable-number tandem repeat.

All data are from healthy adult subjects unless otherwise specified.

a Participants with schizophrenia.

b Participants with major depressive disorder.

c Participants with traumatic brain injury.

d Participants with 22q11 deletion syndrome.

e rs1079597 and rs1076560 are in linkage disequilibrium (*r*^2^=0.87, *D*′=0.93, northern and western European ancestry).

f Participants with tobacco dependence, otherwise healthy.
